# The discursive (re)construction of social relations in a crisis situation: A genre analytical approach to press conferences on COVID-19 in China

**DOI:** 10.3389/fpsyg.2022.991813

**Published:** 2022-11-08

**Authors:** Hong Wang, Yunfeng Ge

**Affiliations:** School of Foreign Languages, Shandong Normal University, Jinan, China

**Keywords:** social relation, critical genre analysis, critical discourse analysis, press conference, COVID-19

## Abstract

The outbreak of COVID-19 has imposed a great threat both to people’s health and to social relations. By following the theoretical constructions of critical genre analysis and critical discourse analysis and drawing on the 35 press conferences on the COVID-19 outbreak in China, this paper explores how the discourse of press conferences is used by the Chinese government as a means of crisis management to (re)construct social relations. The analysis of the data reveals a hybridity of social relations reproduced discursively between such social actors as government institutions, the general public, medical institutions or staff, and COVID-19 patients, and a distinct feature of interdiscursivity of the discourse of press conferences on COVID-19, with descriptive, instructional, strategic, and evaluative discourses being the most frequently employed. It is also found that political, professional, social, and cultural forces are interwoven with each other to contribute to the interdiscursivity of the discourse of press conferences and the hybridity of social relations constructed thereof.

## Introduction

Since its outbreak in early 2020, the COVID-19 pandemic has constituted not only a threat to public health, but also an economic and social crisis. In the face of the destabilized social order, uncertainty about the severity of the pandemic, and unpredictable recession of the economy, people are confronted with a social crisis with social structures broken down and relationships between people affected (Jonas and Morton, 2012). As a result, crisis management and resolution are not only a matter of material actions but also “crucially depend for their successful performance on language use, communication, and discourse” (De Rycker and Mohd Don, 2013, p. 3).

While taking material actions to contain the pandemic, governments, and institutions have also attached great importance to the semiotic properties of the crisis, deploying various forms of news media to communicate to the public the progress in the prevention and control of the pandemic (Ruson and Diamant, 2022; Tang and Lee, 2022). These intended communications are expected to play a constitutive role in the successful management and resolution of the crisis event. Among the various forms of communication, press conferences are usually regarded as the most preferred for their immediacy in information sharing, and interactive, dynamic, and collaborative features (Yi and Chang, 2012).

This study is devoted to an analysis of the discursive (re)construction of social relations by the Chinese government through the discourse of press conferences on COVID-19 in China. In analyzing the dialectical relationship between discourse and society, we take an action orientation perspective and understand discourse not simply as a means to retrieve a stock of information, but as a set of resources employed by its users to accomplish a wide variety of social actions (*cf.* Krippendorff and Halabi, 2020). In light of this understanding, this study is based on the following assumptions: (1) the COVID-19 crisis is not only a material and empirical reality, but also a socially constructed phenomenon; (2) discourses play a crucial role in the construction and constitution of the social reality of the COVID-19 crisis; and (3) press conference is a generic type of discourse used by governments as a means of intervention to (re)construct the social relations that are affected by the COVID-19 crisis.

Based on the above assumptions, this study is aimed at addressing the following three research questions: (1) What social relations does the Chinese government endeavor to (re)construct through the discourse of press conferences on COVID-19 in China? (2) What discursive strategies are employed in these press conferences to (re)construct these social relations? (3) What social factors contribute to the discursive features of these press conferences? It is hoped that this study will contribute to our understanding of how press conferences are used by the Chinese government as an institutionalized intervention to the COVID-19 crisis, what social relations gain prominence in the discourse of press conferences and how these relations are reproduced discursively.

## Literature review

### Crisis situation

Crisis usually refers to a “major occurrence with a potentially negative outcome” (Fearn-Banks, 2011: 2), which may “create high levels of uncertainty and simultaneously present an organization with both opportunities for and threats to its high-priority goals” (Seeger et al., 1998: 239). Crises are regarded by many as having not only an objective or systemic aspect, but a crucial subjective aspect as well in that, in a crisis situation, people have to make decisions about how to develop strategies for pursuing particular courses of action or policies to restore balance and rationality (Fairclough and Fairclough, 2013: 3). Although crisis literature abound in studies on the objective aspect, for example, definitions, causes, consequences, etc. of crises (i.e., Sellnow and Seeger, 2021), many accounts of crises recognize the importance of their subjective aspect.

Studies on the subjective aspect of crises emphasize that both people’s understanding and the outcome of crises depend on how crises are symbolized or constructed. For example, Žižek (2009) emphasizes that ideological interpretation or the narrative of crisis imposes itself and determines the general perception of the crisis. Jessop (2002: 94) holds that crises create the space for competing “strategic interventions to significantly redirect the course of events.” Therefore, it follows that crisis management is to a large degree a matter of crisis communication which involves “discursive struggles between different narratives of the nature, causes and significance of the crisis and how it might be resolved” (Fairclough and Fairclough, 2013: 3).

Another research topic concerning the subjective aspect of crises is the agency of different social actors in communication, decision-making, and policy-development in response to crises. While there are still disputes over whether crises should be approached from an organizational perspective or political perspective, researchers recognize the crucial role government plays in both crisis communication and crisis management, especially in such worldwide pandemic as COVID-19 (Tench et al., 2023). Recent studies have highlighted not only how information about crises is collected, analyzed, and disseminated through both traditional and new media, but also how people’s perception of crises is shaped as a result of government’s strategic communication (Coombs, 2015; Lilleker et al., 2021; Tench et al., 2023). For example, Edelman (1977) investigates how political discourses such as presidential addresses in times of crises can be significant in shaping public opinion of the crisis and its management by the authorities. De Rosa and Mannarini (2020) explore the polemical and polarized social representations of COVID-19 pandemic in discourses of Italian government and institutions, and how these pluralistic representations orient people’s feelings and behavior and the construction of the “invisible other.” The study of Sandaran and De Rycker (2013) demonstrates how President Bush, through various legitimating discourses and strategies, exploited the national crisis of September 11 to legitimate and implement government agenda.

All of these studies contribute to useful insights into our understanding of the subjective aspect of crises and the role of government in narratives, explanations, as well as imaginaries of crises, and how these subjective constructions may finally influence crisis management. An essential but insufficiently investigated topic in these accounts is the social relation aspect of crises. The symbolic construction of crises concerns not only the shaping of people’s perception of crises but also the relationship between social actors in the crisis situation who may have different or even conflicting perceptions of crises. Therefore, apart from government’s strategic communication of crises, investigations should also be focused on how government endeavors to negotiate social relations to build trust between different social actors, the public’s trust in government in particular, to influence their intentions to adopt government-recommended guidelines (Blair et al., 2017; Vinck et al., 2019).

### Social relations

As one of the fundamental concepts of sociology, social relation is usually understood as networks people establish with each other during their social activities. Studies on social relations have been conducted from a variety of perspectives, focusing on their different dimensions and aspects.

The psychological perspective regards social relations as a cognitive construal and establishes a dialectic relationship between social relations and people’s mental and physical health (*cf.* Finch and Graziano, 2001; Cacioppo et al., 2010; Ford et al., 2011; Tay et al., 2013; Turner and Turner, 2013). The exploration of the psychological side of social relations has helped to explain individuals’ psychological processes in terms of their social interactions. However, an overemphasis on the psychological side of social relations may lead to the illusion that interpersonal relations are in nature driven and constituted by individual desires and thus neglect their social mechanism.

Other researchers have laid emphasis on the social mechanism of interpersonal interactions and inquired into the social processes that give rise to the various types of social relations and social structures. For example, Heaney and Israel (2008) put forward a framework for the analysis of social relations by classifying social functions into social capital, social influence, social undermining, companionship, and social support, which can be further analyzed on such levels as reciprocity, intensity, homogeneity, etc.

Recent studies have also concerned the relationship between social relations construction and crisis situations or legitimacy threatening events. Researchers have demonstrated that social actors’ communication strategies, media usage, and techniques in crisis situations have a profound effect on public relations. For example, Taylor and Perry (2005) illustrate how timely, accurate, and effective online communication contributes to the restoration of public relations in crisis situations. Lu (2008) demonstrates that fantasy themes of character, action, and setting in the coverage of China’s anti-SARS campaign may lead to a positive portrayal of national leaders, the heroic acts of medical workers, and a renewed sense of nationalism, which in turn can provide moral legitimacy for the new leadership in the time of political transition.

These studies, by relating social relations to individuals’ health, social functions, and crisis situations, help explain the psychological and social factors that give rise to the various types of social networks and structures. However, they are still inadequate in at least two respects. Firstly, they do not incorporate investigation of how social relations interact with the institutions or social practices that produce them, and therefore fail to examine the professional and cultural contexts that help to generate specific types of social relations. Secondly, previous studies fail to recognize the fact that social relation is regarded by governments or institutions as an object of (re)regulation through discourse, especially in the background of a crisis situation.

Therefore, a thorough investigation of how social relations are (re)constructed in a crisis situation cannot be achieved without considering the constitutive power of language, especially the use of certain types of discourses in relation to specific situations or social practices.

### Press conferences

The press conference is a form of public communication organized usually by governments or institutions in relation to specific tasks, practices, and preallocated participant roles (Ekström, 2015). Press conferences have mostly been investigated from the perspectives of political science and journalism. The political science perspective regards press conferences as governmental institutions’ repertoire to influence the public opinion (Ekström, 2015), and as a means of management of public relations (*cf.* Ekström, 2009; Eshbaugh-Soha, 2013; Yi, 2016a). On the other hand, the journalistic perspective regards press conferences as an arena to display journalism’s autonomy and exclusive rights to scrutinize (Ekström, 2006; Clayman et al., 2007; Eriksson and Östman, 2013).

Researchers have also investigated the communicative nature of press conferences. The rhetoric studies on press conferences have focused on the communicative strategies employed by the two sides of press conferences, including interruptions, jokes, negative interrogative structure, etc. (*cf.* Ekström, 2009; Clayman et al., 2010; Heritage and Clayman, 2013). For example, Yi (2016b) investigates the rhetorical structure of the Chinese Premier’s Press Conference and interprets how it evolves from habitualization, objectification, and sedimentation to the present semi-institutionalized form. Strunck (2008) analyzes how rhetorical strategies are employed by politicians in press conferences to gain consensus and thus to convince the audience and the public about the international work for water.

The linguistic studies have investigated how positions and attitudes are represented by different linguistic resources or discursive strategies in press conferences. For example, Bhatia (2006) has found that such linguistic devices as juxtaposition of opposites, sentence initial hedges, modal verbs, technical vocabularies, etc. are used by politicians to conceal reality and accomplish joint goals of diplomacy. Eriksson (2011) explores how follow-up questions are used by journalists to pursue evasive answers to perform both adversarial and non-adversarial actions. Focusing on the interpreter-mediated Premier-Meets-the-Press conferences held annually in China, Gu (2019), by drawing on Bakhtin’s concept of dialogized heteroglossia, explores the discursive strategies interpreters employ to render journalists’ questions in an attempt to (re)construct the Chinese government’s image.

These studies have contributed to our understanding of press conferences as an arena where a variety of social, political, journalistic, and linguistic forces converge to create a hybridity of ideas, social practices, and relations. However, their findings are far from satisfactory for the following reasons. Firstly, previous studies usually regard press conferences as a form of two-party communication between politicians and journalists, without paying attention to the diverse relationships constituted at the same time between other social actors. Secondly, the examination of the underlying forces of press conferences is limited to political and journalistic aspects, without taking into account other socio-cultural forces that contribute to the complexity of press conferences. Thirdly, the linguistic analysis of press conferences is limited to the description or explanation of the rhetoric strategies employed by specific parties, without noticing the constitutive power of the discourse of press conferences and how social realities are (re)constructed and intervened through press conferences.

## Research methodology

### Theoretical framework

This study employs the theoretical constructions of critical genre analysis (CGA) and critical discourse analysis (CDA) to explore how the Chinese government endeavors to (re)construct social relations through the discourse of press conferences in the crisis situation caused by the COVID-19 virus. Over the past decades, our understanding of genre has evolved from simple categorizations of text types to ways of recognizing, reacting to, and helping reproduce recurrent situations (Bawarshi and Reiff, 2010). As one of the discourse analytical approaches, genre analysis focuses on the study of situated linguistic behavior in institutionalized academic or professional settings, by offering “descriptions of language use, incorporating, and often going beyond, the immediate context of situation, and taking analyses beyond mere linguistic descriptions to offer explanation for specific uses of language in conventionalized and institutionalized settings” (Bhatia et al., 2008, p. 10).

Bhatia (2017) has put forward a multi-perspective framework for critical genre analysis (CGA), incorporating textual, socio-pragmatic, and professional spaces. This framework, with its focus on professional practice, is aimed to explore professional practices or actions in typical academic and professional contexts by extending genre theory beyond the analyses of textual, intertextual and other semiotic resources used in professional genres (Bhatia, 2017). The key notions in CGA are criticality, contextualization, and interdiscursivity.

The notion of criticality in CGA seeks to uncover how expert professionals construct, interpret, and exploit genre conventions in the performance of their professional tasks, and how institutional, organizational, and professional activities are constructed, and specific communicative objectives are accomplished.

Contextualization emphasizes that genre analysis should be conducted to account for not only how genres are constructed but also how they are interpreted and exploited in specific professional contexts to achieve specific disciplinary objectives (Bhatia, 2004). In framework of CGA of Bhatia (2017), context is analyzed as three overlapping spaces, namely, textual, socio-pragmatic, and socio-cultural spaces, where discourse operates on the levels of text, genre, professional practice, and professional culture. Investigations of textual space focus on lexico-grammatical resources, and patterns of discoursal and rhetorical structures within the context of generic conventions and practices. Investigations of socio-pragmatic dimensions of space highlight analysis of social structure, interactions, goals of the professional community, practitioner guidance, etc. not only in the process of genre construction but also in audience reception procedures and insights. Investigations of socio-cultural space focus on the constraints of professional culture, professional or corporate identities, institutional changes, etc. on the construction and interpretation of genres.

Interdiscursivity is defined by Bhatia (2010, p. 35) as the “innovative attempts to create various forms of hybrid and relatively novel constructs by appropriating or exploiting established conventions or resources associated with other genres and practices.” It highlights the fact that professional communication is a discursive space where different socio-cultural, institutional, and organizational dynamics are negotiated and where different generic resources and semiotic modes of communication are appropriated to achieve professional objectives (Ge and Wang, 2019). Interdiscursivity helps to account for a variety of discursive processes and professional practices, such as the mixing, embedding, and bending of generic norms in professional contexts (Bhatia, 1993, 2004, 2010). Moreover, by incorporating the concepts of dynamism, hybridization, and innovation to reflect the evolution of genres, interdiscursivity helps to account for the polyphonic and heterogeneous features (Bakhtin, 1981) of a specific type of discourse, and, more importantly, the social stratification that gives rise to the heterogeneity of that type of discourse.

Critical discourse analysis shares with CGA the basic assumption that on most occasions social events or practices are discursively shaped and enacted. While both approaches take a critical perspective toward discourses in an attempt to reveal the hidden underpinning socio-cultural forces or power relations, they put emphasis on different aspects of the concept of criticality. CGA regards language use as primarily a professional practice and therefore its main strengths lie in interpreting the relation between professional culture or community and generic resources and in explicating how specific genres of discourses are exploited to achieve specific disciplinary objectives in professional settings and. CDA, on the other hand, takes language use as a social practice and therefore focuses more on the macro-relationship between language, power, and ideology, and attempts to redress the asymmetrical power imbalances that impede social, political, and cultural processes through the analysis of semiotic data (Fairclough, 1992).

The reason why we combine CGA and CDA is that, by putting discourses which are accessed from a professional perspective of CGA into a broader social, cultural, and political background, the newly built framework can help us to have a more profound investigation of the power and force underlying the processes of how genres are formed or mixed to construct social realities. As far as this study is concerned, the combination of CGA and CDA is expected to achieve a more fruitful understanding not only of the generic features of the discourse of press conferences on COVID-19 in China and the discursive strategies that are employed by the Chinese government in an attempt to construct different social relations among various social actors, but also of the underpinning social, cultural, political, and professional background and ideologies that contribute to the hybridity or interdiscursivity of the discourse of press conferences.

In this study, the notion of criticality, which is aimed at explicating how social actions or professional objectives are achieved through linguistic and generic resources, contributes to the exploration of how and why the Chinese government intervenes the social crisis caused by the COVID-19 pandemic and, in particular, how the Chinese government (re)constructs social relations through the discourse of press conferences. The notion of contextualization helps to analyze the reciprocal dynamic between lexico-grammatical recourses, discursive strategies, rhetorical devices, etc. of press conferences and the different layers of context constituted as socio-pragmatic and socio-cultural spaces, where such dynamics as social relations, professional identities, goals of communication, etc. contribute to the production and reception of press conferences. The notion of interdiscursivity helps to explore the hybridity of the discourse of press conferences, and, more importantly, how different genres are employed and mixed by the Chinese government in the discourse of press conferences in an attempt to (re)construct a diversity of social relations as a response to the COVID-19 crisis.

### The data

The data used for this study consist of 35 official press conferences, among which 21 were held by the State Council of People’s Republic of China and 14 by the local government of Hubei Province (Wuhan is the capital city of Hubei Province). Each press conference usually had three to four spokespersons, including a host, one to two officials who are from the local government of Hubei Province or Chinese National Centre for Disease Control and Prevention, and sometimes an infectious disease specialist. These press conferences were attended by media from print, television, radio, and the internet. The press conferences analyzed in this study were recorded when they were broadcast live on the internet from January 23 to April 8, 2020, during which period the city of Wuhan was locked down. There are two reasons for using the data: Firstly, these press conferences were the first official occasion where the COVID-19 virus was discursively represented after its breakout. The analysis of the data may contribute to our understanding of how the Chinese government perceived the virus at its early stage of development. Secondly, it is in this period of time when China suffered the most severe impact of the COVID-19 pandemic. Therefore, the investigation of the data is expected to have a better demonstration of how the Chinese government intervenes the social crisis through the discursive (re)construction of social relations.

The recordings of the press conferences were first transcribed with the help of computer software and then proofread by the authors of the study to ensure their accuracy and faithfulness. Altogether, 32 h and 45 min of video were captured, which were transcribed into a corpus of 370,601 Chinese characters. The examples used in the paper were translated into English by the authors of the study.

Based on the above theoretical constructions of CGA (Bhatia, 2017) and CDA (Fairclough, 1992), this study first identifies the generic structure of the press conferences on the COVID-19 pandemic in China, the social actors involved, and the different relations constructed among these social actors. Secondly, the study identifies the different types of discourses employed by speakers of the press conferences for the construction of the social relations. Thirdly, each type of the discourse is examined carefully to demonstrate how both discoursal patterns and lexico-grammatical choices contribute to the discursive construction of the social relations. Finally, attention is focused on the interpretation of how the context of professional identities, values, and the broader social, cultural, and economic background impose a constraint on the discursive (re)construction of social relations.

## Findings and analysis

Based on the above procedure, the analysis of the data has revealed the following findings in relation to the three research questions: Firstly, the press conferences on COVID-19 in China have manifested a four-move pattern (see Table 1): opening statement, report on updated information on COVID-19, question and answer session, and closing statement.

**Table 1:** Generic structure of press conferences on COVID-19 in China.

Move 1	Opening statement Step 1: Introducing main topics of the press conference Step 2: Introducing spokespersons
Move 2	Report on updated information on COVID-19
Move 3	Question and answer session Step 1: Questioning from journalists Step 2: Mediating by the moderator Step 3: Answering from spokespersons
Move 4	Closing statement Step 1: Informing about next press conference Step 2: Concluding the press conference

Keyword analysis of the data has highlighted four groups of social actors in the COVID-19 crisis, namely, government institutions, the general public, medical institutions or staff, and COVID-19 patients. Table 2 has shown the top seven keywords of each group of social actors and their number of occurrences. It has been found that, out of these social actors, four major types of social relations are reproduced in these press conferences, centering around government institutions: relation between government and the general public, relation between government institutions of different levels, relation between government and medical staff, and relation between government and COVID-19 patients. The social actors and their relations are mainly distributed and constructed in Move 2 and Move 3 of the press conferences.

**Table 2:** Social actors identified in press conferences on COVID-19 in China.

Government institutions	General public	Medical institutions/staff	COVID-19 patients
CPC Central Committee (262) Local government (157) State Council (145) Headquarters of Prevention (52) General Secretary Xi Jinping (46) Central directing group (26) Health Commission (18)	Companies (697) Wuhan (572) Local community (430) The public (119) Residents (97) All sectors of society (24) Common people (23)	Hospitals (623) Medical teams (210) Expert (groups) (182) Doctors (124) Medical workers (108) Medical institutions (93) Nurses (46)	Patients (502) Sick cases (402) Sick people (382) People of close contact (103) Senior citizens (75) Infected people (26) Children (18)

Secondly, spokespersons at the press conferences have employed a diversity of generic resources to reproduce these social relations, among which four types of discourses are identified as frequently used: descriptive discourse, instructional discourse, strategic discourse, and evaluative discourse, By reporting daily updated information on COVID-19, descriptive discourse is oriented to increasing the public’s trust in the government; characterized with more interventional features, instructional discourse is employed by spokespersons to provide the public and local institutions with guidance for appropriate actions; strategic discourse is mainly used to promote the central government’s management measures on the prevention and control of the pandemic, thus reproducing the hierarchical relation between the central government and local governments and institutions; and by drawing on such parameters as importance, emotivity, etc., evaluative discourse highlights the contributions and devotions of specific institutions or groups of people during their fight against the COVID-19 pandemic.

Thirdly, the hybridity of the professional genres employed at the press conferences can be attributed to several contextual factors, where the dynamics of professional, political, social, and cultural forces are interwoven with each other to contribute to the heterogeneity and complexity of social relations.

### Descriptive discourse: Reporting updated information

The analysis of the data suggests that the reporting of the updated information on COVID-19 is realized through descriptive discourse, with which spokespersons cite specific figures and statistics to report the ongoing pandemic situation in China. With its assumed objectivity and neutrality, the sharing of information through descriptive discourse can not only remove people’s anxiety about the coronavirus, but, more importantly, help to reproduce a government-public relation based on trust and understanding, as illustrated by the following example.

Extract 1 (January 28, 2020, State Council of PRC).

(1) 截至1月28日24时，国家卫生健康委收到各省 (区、市)累计报告确诊病例5,974例，(2) 现有重症病例1,239例，(3) 累计死亡病例132例，(4) 累计治愈出院103例，(5) 现*有*疑似病例9,239例，(6) *有*59,990人正在接受医学观察.

(1) Up to 24:00 28th January, the National Health Commission *has received* from all the provinces (regions and municipalities) a cumulative report of 5,974 confirmed cases of COVID-19. (2) Up to now, *there are* 1,239 patients in severe condition. (3) The number of deaths *has added up* to 132. (4) The number of patients who are discharged from the hospital *has added up* to 103. (5) Up to now, *there are* 9,239 suspected cases. (6) *There are* 59,990 people who are under medical observation for close contact with confirmed cases.

Extract 1 is from the press conference held by the State Council of the PRC on January 28, 2020. In this extract, descriptive discourse is used to report to the public the latest information on the COVID-19 in China. The sudden outbreak of the COVID-19 has aroused the public’s doubts about Chinese government’s transparency in information disclosure and concern about the severity of the pandemic. The press conferences have offered an arena to increase the public’s trust in the government by immediate disclosure of information.

The sharing of the information is characterized by the employment of statistical figures and the objective tone of voice, which help add to the accuracy of the information. These statistical figures, which cover six different groups of people ranging from patients of confirmed cases to people under medical observation, demonstrate to the general public the government’s rigorous attitude towards the pandemic.

Moreover, in order to ensure the objectivity of the information, declarative sentences are employed to give a detailed elaboration of the information. For example, the existential structure “有(there are)” in clauses (2), (5), and (6), and the statistical term “累计(has added up)” in clauses (3) and (4) report the numbers of patients in severe condition, people of suspected cases, people under medical observation, etc. They give the impression that the events concerning these different groups of people happened naturally, thus enhancing the objectivity of the information. These declarative structures and statistical figures, when ensuring the objectivity of the shared information, help to improve the government’s trustworthiness, and thus contribute to the construction of a trust relation between the government and the general public.

### Instructional discourse: Providing guidance for appropriate actions

Instructional discourse, which is commonly seen in specialist contexts, is usually employed by experts to instruct people on “how to perform a specialized sequence of activities in relation to certain objects and locations” (Martin and Rose, 2008, p. 182). Instructional discourse usually involves the teaching of certain procedures or skills through experts’ illustrative performance of target activities. Consequently, it usually constructs a hierarchical relation between experts and the target group of people due to the imbalance of knowledge.

The analysis of the data has shown that, at the press conferences on COVID-19 in China, instructional discourse is mainly used to instruct the public to behave appropriately to avoid being infected by the virus. Given usually by spokespersons with medical expertise, these instructions help to construct a government public relation based on expert knowledge, as illustrated by the following example.

Extract 2 (February 6, 2020, Hubei Province).

(1)居民*需要*做好以下几方面工作:(2)首先是加强居家通风与勤戴口罩…；(3)第二是尽量*不要*出行，*不*到人群密集，通风不良的场所…；(4) 第三是注意咳嗽礼仪和手卫生，(5)咳嗽、打喷嚏时*应该*用纸巾遮掩口鼻…；(6)第四，…共用餐具*需*采用一人一套的原则；(7)第五，*要*注重营养，睡眠，加强居家体育锻炼，增强自身免疫力…

(1) Residents *shall* pay close attention to the following aspects. (2) First, strengthen indoor ventilation and wear face masks… (3) Second, try *not* to travel, and do *not* go to crowded areas or places with poor ventilation… (4) Third, mind respiratory etiquette and hand hygiene. (5) Cover mouth and nose with tissues when coughing or sneezing… (6) Forth, follow the principle of individual serving when dining together… (7) Fifth, pay close attention to nutrition and sleep, and enhance indoor physical exercises to strengthen self-immunity…

In Extract 2, which is from the press conference held by Hubei Province on February 6, 2020, Tong Yeqing, an official medical expert from Research Institute for Communicable Disease Control and Prevention of Hubei CDC, elaborates on the measures people should adopt to avoid being infected by the virus. The instructional features are first embodied in the procedural structure of the discourse, where ordinal numerals are employed by the spokesman to present the different steps people should take to implement self-protection. On the grammatical level, all the five instructions are incorporated in imperative structures, indicating compulsion and necessity. For example, in clause (1), the topic sentence of the text, the employment of the modal auxiliary “需(shall)” makes a clear signal that what the spokesman is going to talk about are requirements imposed on the residents. In the following six imperative structures, the imperative verbs such as “加强(strengthen),” “注意(mind),” “遮掩(cover),” etc. make the utterances more like demands than instructions. In particular, the prohibition of behaviors expressed by the negative polarity adjunct “不要/不(not)” in clause (3) constitutes a warning and thus presents more binding forces on people’s outdoor activities.

Through Extract 2, the spokesman of the press conference has helped to construct a government public relation based on his different access to expertise in coronavirus prevention. Different from Extract 1 where the government functions to disclose information, in Extract 2 the government functions to give advice to the public on how to behave properly during the pandemic, thus constituting a hierarchical relation between government and the public. The construction of this hierarchical relation is mainly attributed to the spokesman’s expert knowledge in novel coronavirus and the corresponding authority in instructing the public on the pandemic prevention and protection.

On other occasions, a hierarchical relation is established by instructional discourse between the central government and local authorities or institutions. Rather than given as advice, these instructions are usually presented with more administrative and coercive characteristics, and thus demonstrate more binding forces, as illustrated by the following example.

Extract 3 (February 7, 2020, State Council of PRC).

(1)督促湖北省和武汉市依法采取最严格防控措施，加强农贸市场监管和野生动物管控。…(2)要准确、及时地追踪到密切接触者，…(3)同时需要基层社区组织、基层医疗卫生组织和疾病预防控制机构密切配合。(4)对于…密切接触者，发现以后应实行集中医学隔离。(5)对不具备条件的地区，可以采取居家隔离医学观察。(6)如果密切接触者拒不执行，根据传染病防治法的规定，可以由剬安机关协助采取强制措施.

(1) Hubei Province and Wuhan Municipality are urged, *in accordance with the law*, to take the strictest measures of prevention and control to enforce the supervision of farmers’ markets and the management and control of wild animals… (2) People of close contact *shall* be traced accurately and timely… (3) Meanwhile, grassroot community organizations, grassroot medical organizations, and institutions for disease prevention and control *shall* cooperate closely. (4) As for people of close contact, concentrated quarantine *shall be* conducted after they are found. (5) In those regions where conditions do not permit, indoor quarantine and medical observation *can* be conducted. (6) Where people of close contact refuse to cooperate, coercive measures *can* be adopted by the public security organ according to Law on the Prevention and Control of Infectious Diseases.

Compared with Extract 2, the instructions in Extract 3 assume more binding forces and coercive characteristics, based on which an administrative relation between the central government and the subordinate local governments and institutions is constructed. On the lexical level, the employment of the causative verb “督促(urge)” in clause (1) makes the text more like an order rather than an instruction. On the syntactic level, the spokesman employs the modal auxiliary verb “要(shall)” in clauses (2), (3), and (4) to indicate duty and obligation. The permissive auxiliary verb “可以(can)” in (5) and (6), which means permission and approval and is less binding compared with “要(shall),” also contributes to the construction of the administrative relation in that it indicates an authorization from the central government.

It can be seen that, by employing causative phrases, compulsory structures and permissive auxiliary verbs, Extract 3 demonstrates a distinct feature of legal discourse, which constitutes the phenomenon of “genre embedding,” where text-external semiotic resources across genre conventions are appropriated for the purpose of “private intentions” (Bhatia, 2004, 2017). The embedding of the legal discourse here in the instructions can be further demonstrated by the spokesman’s citation of laws in (1) and (6) with the circumstantial adjuncts “依法 (in accordance with the law)” and “根据传染病防治法的规定 (according to Law on the Prevention and Control of Infectious Diseases).” Moreover, the employment of the formal and technical terms such as “集中医学隔离 (concentrated quarantine),” “医学观察(medical observation),” etc. also help to increase the binding force of the instructions on local institutions.

### Strategic discourse: Promoting management measures

Strategy refers to the specific function of management with a particular role within organizations to act, plan, and think about how they work, survive, and prosper (Morgan and Sturdy, 2000). In general, strategies are constructed, enacted, and reproduced as a discourse, which comprises those “actions, interactions, and negotiations of multiple actors and the situated practices that they draw upon in accomplishing that activity” (Jarzabkowski et al., 2007, p. 8). Strategic discourse usually functions as the medium through which senior management make sense of, respond to and thereby help to cope with the developments or changes of the organization and its context. Intimately related to power within organizations, strategic discourse has become an arena where senior management both legitimate their existence and structure their actions (Marglin, 1974). Therefore, while highlighting the primary role of the leadership of organizations, strategic discourse constructs a hierarchy between strategy-makers and mundane, operational and functional executors of the strategy.

The discourse of press conferences on COVID-19 in China manifests a distinct strategic feature, where management decisions and operational measures are integrated to effect the prevention and control of the pandemic, and by so doing to construct a more complicated hierarchical relation between institutions at different levels, as illustrated by the following example.

Extract 4 (February 15, 2020, State Council of PRC).

(1)新型冠状病毒肺炎疫情牵动着全党全国人民的心。(2)党中央国务院高度重视，(3)习近平总书记多次主持召开重要会议，对疫情防控工作做出全面部署。……(4)国家卫健委迅速行动，提请国务院将新冠肺炎纳入了传染病防治法乙类传染病，……(5)各省份都迅速地建立了联防联控机制，(6)武汉市……加强了患者的救治。……(7)经过不懈的努力，……有效地压低了流行高峰，削弱了流行的强度.

(1) (BACKGROUND) The novel coronavirus pandemic has been affecting the hearts of the Party and people all over the country. (2) (MANAGEMENT DECISION) The CPC Central Committee and the State Council have attached great importance to the pandemic. (3) (MANAGEMENT DECISION) General Secretary Xi Jinping has presided over several important meetings to make overall arrangements for the prevention and control of the pandemic… (4) (OPERATIONAL MEASURE) *The National Health Commission* has moved *quickly*, and submitted to the State Council for approval to classify the novel coronavirus pneumonia as a category B infectious disease under the Law on Prevention and Control of Infectious Diseases… (5) (OPERATIONAL MEASURE) *All provinces* have *promptly* established joint prevention and control mechanism. (6) (OPERATIONAL MEASURE) *Wuhan Municipality*…has strengthened the treatment of the patients… (7) (RESULT) After persistent efforts, the peak of the pandemic has been effectively lowered, and its intensity has been reduced.

The strategic discourse in Extract 4 demonstrates a distinctive generic structure, comprising of the moves of Background, Management Decision, Operational Measure, and Result. The Background move in clause (1) introduces the public health crisis caused by the novel coronavirus. The following two clauses, (2) and (3) constitute the move of Management Decision, where decisions from top-level administration, namely, “党中央国务院 (The CPC Central Committee and the State Council)” and “习近平总书记 (General Secretary Xi Jinping),” are introduced. The move of Operational Measure, which comprises of clauses (4), (5), and (6), describes the specific measures that are adopted by lower-level institutions. Clause (7), the move of Result, emphasizes the positive effect of the management decisions and operational measures.

On the syntactic level, both moves of Management Decision and Operational Measure are realized by material processes. The “Actor-Process-Goal” structure, apart from reporting the decisions and measures for combating the pandemic, demonstrates the hierarchical relation between different actors of the processes. Namely, the operational measures taken by the National Health Commission, all provinces, and Wuhan Municipality in (4), (5), and (6) are regarded as responses to the decisions of the CPC Central Committee, the State Council, and President Xi Jinping. The hierarchical relation is further highlighted by different lexical devices. For example, the binding force of management decision in (2) and (3) is demonstrated by such expressions as “高度重视 (attach great importance to),” “重要会议 (important meeting),” and “全面部署 (make overall arrangement),” which are usually used in solemn, authoritative, and policy contexts in China. In contrast, the responding actions of subordinate institutions in (4), (5), and (6) are defined by the spokesman as being “迅速 (quickly)” and “迅速地 (promptly).”

Embedded in a narrative tone, the decisions and measures in Extract 4 are presented as happening in the past space. On other occasions, strategies and operational measures are introduced in the future space as plans or policies, which, while expressing expectation and determination, contribute to the construction of the relation between the government and people of specific groups, especially those COVID-19 patients, as illustrated by the following example.

Extract 5 (January 22, 2020, State Council of PRC).

(1)三是*全力*救治患者。(2)调配*最强的*中西医疗资源和专家资源，中西医结合，*最大限度减少*死亡病例。(3)加强患者医疗救治费用保障，确保患者得到*及时救治*。(4) 相信我们有*以习近平同志为核心的党中央坚强领导*，*有中国特色社会主义的制度优势*，……我们有信心取得疫情防控的胜利.

(1) (OPERATIONAL MEASURE) Third, *all efforts* shall be exerted to treat and cure patients. (2) *The strongest* resources of traditional Chinese and western medicine and specialists shall be arranged, combining traditional Chinese and western medicine treatments, to *furthest minimize* death cases. (3) The coverage of the medical expenses incurred by patients shall be safeguarded to ensure the *timely medical treatment* of the patients. (4) (DETERMINATION) We believe that, *under the strong leadership of the CPC Central Committee with Xi Jinping at the core*, and *with the institutional advantages of socialism with Chinese characteristics*, …we have confidence to achieve victory over the pandemic.

Compared with strategic discourse as implemented decisions or measures, strategic discourse as plans or policies mainly functions to express government’s expectation and determination, and is oriented toward people of specific groups, such as patients, medical staff, volunteer workers, etc. Extract 5 addresses patients infected with the novel coronavirus and, by publishing the detailed treatment plan, demonstrates the government’s determination to treat and cure those patients.

On the generic level, clauses (1), (2), and (3) constitute the move of Operational Measure, and clause (4), the move of Determination. While disclosing the specific treatment measures, the three clauses in the move of Operational Measure employ empathetic structures beginning with such verbs as “救治 (treat and cure),” “调配 (arrange),” and “加强 (safeguard)” to express the government’s determination to treat the patients. Moreover, the determination is further strengthened by such expressions as “全力 (all efforts),” “最强 (the strongest),” “最大限度减少 (furthest minimize),” and “及时救治 (timely medical treatment).” In the move of Determination, the government’s determination is demonstrated more explicitly in (4) through the structures “相信 (we believe)” and “我们有信心 (we have confidence).” The circumstantial adjuncts “以习近平同志为核心的党中央坚强领导 (under the strong leadership of the CPC Central Committee with Xi Jinping at the core)” and “有中国特色社会主义的制度优势 (with the institutional advantages of socialism with Chinese characteristics),” which reveal the sources of confidence, add to the force and acceptability of the determination.

### Evaluative discourse: Highlighting contributions and devotions

Evaluation refers to “the expression of the speaker’s or writer’s attitude or stance towards, viewpoint on, or feelings about the entities or propositions he or she is talking about” (Hunston and Thompson, 2000, p. 5). Since people’s attitudes or feelings may concern different aspects of the world, evaluations are usually closely related to socio-cognitive resources (Bullo, 2019) and can be conducted around different value systems through specific stance strategies (Diaz and Shahri, 2020, p. 26). Bednarek (2006) has summarized six parameters to evaluate people’s attitudes: “emotivity (good or bad),” “importance (important or unimportant),” “reliability (genuine or fake),” “expectedness (expected or unexpected),” “comprehensibility (comprehensible or incomprehensible),” and “necessity (necessary or unnecessary).” These parameters can help us understand the complex functions of evaluative discourse on different dimensions.

In the discourse of press conferences on COVID-19 in China, evaluation is usually directed to specific institutions or groups of people, with evaluation resources being used to make comments either on the importance or effects of strategic decisions or operational measures, or on the devotions or contributions of those institutions or people, as illustrated by the following example.

Extract 6 (February 15, 2020, Wuhan Province).

(1)方舱医院在这一次疫情防控当中起到了至关重要的作用。(2)方舱医院是一个效率很高，同时成本很低的一个方法。(3)当前在武汉医疗资源、床位资源还是相当紧张的，(4)方舱医院的启用集中收治了大量的轻症确诊患者，……极大地提高了我们的收治率以及我们的治疗效果。(5)方舱医院现在使用的过程当中起到了一个非常好的效果.

(1) Mobile cabin hospitals have played a role *of vital importance* in the prevention and control of the pandemic. (2) Mobile cabin hospitals are *highly efficient* and meanwhile *extremely low-cost*. (3) At present, there still has been an extraordinary shortage of medical resources and hospital beds in Wuhan. (4) Mobile cabin hospitals have admitted *a large number of* patients of mild symptoms; … and have *greatly* improved the admission efficiency and treatment effect. (5) It can be concluded that the employment of mobile cabin hospitals has achieved *very good* results.

Extract 6 is an evaluative discourse that directly follows the move of Operational Measure of a strategic discourse. The spokesman, after introducing the measures Wuhan has adopted in the prevention and control of the pandemic, expresses his attitudes towards the employment of mobile cabin hospitals. The spokesman’s evaluation is realized mainly through the parameters of “importance” and “emotivity,” demonstrating both the important role mobile cabin hospitals has played and the good results they have achieved during the prevention and control of the pandemic.

In clauses (1) and (4), the spokesman’s attitude is expressed through the parameter of “importance.” The phrase “至关重要 (of vital importance)” in (1) is a general evaluation on the importance of the mobile cabin hospitals in Wuhan. The evaluation on their importance is then further developed by clauses (3) and (4). Clause (3), by describing the shortage of medical resources and hospital beds, creates a contrastive background against which the functions of mobile cabin hospitals become significant. Clause (4) gives a specific illustration of the functions of mobile cabin hospitals. Such expressions as “大量的 (a large number of)” and “极大地 (greatly)” help to intensify the spokesman’s mood of evaluation. In clauses (2) and (5), the spokesman’s position is expressed through the “emotivity” parameter. Such expressions as “效率很高 (highly efficient),” “成本很低 (extremely low-cost),” and “非常好的效果 (very good results)” demonstrate the advantages of the mobile cabin hospitals.

The spokesman’s positive evaluation in Extract 6, on the one hand, proves the importance and advantages of mobile cabin hospitals, and, on the other hand, embodies the central government’s appreciation of the executor of the operational measure, namely, the local government of Wuhan Municipality. On other occasions, government’s appreciation is directed towards specific groups of people such as medical staff, volunteer workers, policemen, etc. for their devotion and effort in fighting against the pandemic, as illustrated by the following example.

Extract 7 (March 21, 2020, State Council of PRC).

(1)疫情发生以来，广大医务工作者*不顾个人安危*，*舍小家为大家*，迎难而上，*英勇奋战*在抗疫最前线，(2)为保护人民生命健康做出了*重大贡献*，(3)我们*表示崇高敬意*.

(1) Since the outbreak of the pandemic, all the medical staff, *regardless of personal safety* and *sacrificing their own families for the interest of the whole country*, have taken on a difficult task and *put up a brave fight* on the front lines of the pandemic. (2) They have made *great contributions* to the protection of people’s lives and health. (3) To them we *pay our highest respect*.

Extract 7 is an evaluative discourse that expresses the government’s gratitude to the medical staff for their contribution and sacrifice in fighting against the pandemic. The three clauses in the text constitute the pattern of “narration-evaluation-appreciation.” With clause (1), the spokesman gives a general narration of the contribution and sacrifice of the medical staff during the pandemic. Despite its narrative function, clause (1) is also embedded with rich evaluative resources. For example, the phrases “不顾个人安危 (regardless of personal safety)” and “舍小家为大家 (sacrificing their own families for the interest of the whole country)” demonstrate the nobility of the behaviors of the medical staff; and the metaphoric expression “英勇奋战 (put up a brave fight)” defines them as fearless warriors.

Compared with the implicit and embedded evaluation in clauses (1), (2) and (3) are more explicit in expressing the spokesman’s attitude and emotion through the parameters of “importance” and “emotivity.” For example, in clause (2) the phrase “重大贡献 (great contributions)” defines the importance of the work of the medical staff; and in clause (3) the phrase “表示崇高敬意 (pay our highest respect)” demonstrates, through the spokesman, the government’s appreciation of the contribution, and sacrifice of the medical staff.

## Discussion

Discourse is never a unitary and abstract grammatical system of normative forms, but instead a multilayered polyphonic entity where a multitude of bounded verbal-ideological and social belief systems operate in the field of force relations (Bakhtin, 1981). This interdiscursive orientation of discourse, which on the linguistic level can manifest itself inside a particular type of discourse, amid different types of discourses, and amid discourses of different cultures or languages, is attributed to the tensions between centripetal and centrifugal forces during the production of discourse which strive to keep the discourse coherent and apart at the same time (Bakhtin, 1981, p. 275). As such, three kinds of dialogic phenomena can be identified functioning at the same time during the production of discourse at three different levels: the internal dialogism inside discourse, the external social heteroglossia of different belief and value systems, and the mutual interaction between discourse and social heteroglossia. With each type of discourse carrying its own particular points of view, the dialogic phenomena give rise to the stratification of language in discourse, which may further contribute to such interdiscursive phenomena as genre mixing, genre embedding, or genre colonization. Therefore, in essence, discourses are heterogeneous, dynamic, and fragile, and can be broken into pieces and combined by overlapping, mixing, blending, etc. to form new types of discourses where different ideologies, beliefs, and values predominate.

On the other hand, discourse does not simply represent the world, but instead constantly impacts on the world through the way in which its practitioner uses or reproduces meaning to frame the world. Interdiscursivity or heterogeneity of discourse is the consequence of one generic type of discourse performing different kinds of actions at the same time promoted by different or even conflicting values or beliefs. In the case of the discourse of press conferences on COVID-19 in China, its interdiscursive feature is, firstly, a kind of internal dialogism as a direct result of spokespersons’ employment of different types of discourses; secondly, the consequence of Chinese governments’ effort to restore the social relations impaired by the pandemic; and thirdly, the interaction between the interdiscursive discourse of press conferences and social heteroglossia of different belief and value systems.

As a kind of official discourse to manage the social crisis caused by the pandemic, press conferences on COVID-19 in China have been employed by the Chinese government as a mediational means to (re)construct a variety of social relations centering around government institutions. Among them, the relation between government and the public, the administrative and hierarchical relation between different government institutions, and the relation between government and particular groups of people such as medical staff and patients are the most predominant. Underlying these relations are the diverse and sometimes conflicting forces embodying different intentions and ideologies engendered by the political, and socio-cultural contexts in China. These forces, which are centripetal on the side of the government and centrifugal on the side of specific institutions and groups of people, contribute to the diversity of the social relations and the interdiscursivity of the discourse of the press conferences on COVID-19 in China.

Among these relations, the relation between government and the public is always regarded as the most fundamental and important, in that the Chinese government alleges to take as its creed “to exercise power for the benefit of people.” The (re)construction of government-public relation in the press conferences is realized mainly through descriptive and instructional discourses. Descriptive discourse is frequently used to share updated information about the pandemic. Addressed to the general public and with its objective tone, descriptive discourse is embedded with the ideology that the information about the pandemic shared by the government is authentic and trustworthy. Also addressed to the general public, instructional discourse assumes more imperative and technical features in introducing the self-quarantine requirements the Chinese government imposes on its people. With these instructions, the Chinese government attempts to convince people that self-quarantine is the most effective way to prevent the spread of the virus.

The treatment of the COVID-19 patients is another focus of the press conferences. Spokespersons usually resort to strategic discourse to illustrate with specific measures the efforts that the government has made in treating and curing the patients. The discursive construction of the relation between government and the patients through strategic discourse may help to improve the image of the government since it may be regarded as a demonstration of the “people-oriented doctrine” and the “serve-the-people creed.”

The relation between government and medical staff is constructed mainly through evaluative discourse. By depicting them as soldiers and “the most lovable people of the new era,” spokespersons express government’s appreciation for the contribution and sacrifice of the medical staff. Being inspiring and encouraging, government’s appreciation is aimed, on the one hand, to enhance the internal cohesion and the centripetal force of the country, and on the other, to increase people’s confidence in fighting off the coronavirus.

Compared with other types of relations, the relation between government institutions is more complicated due to their hierarchical characteristics. On the one hand, the authority of the CPC and the central government is displayed by strategic discourse and instructional discourse where the central government makes policies or gives instructions that are binding on governments or institutions at lower levels. On the other hand, the strategic discourse oriented to governments at lower levels is of more operational and executive feature, where local governments or institutions are depicted as determined in carrying out the central government’s policies. The hierarchical relation is also embedded in evaluative discourse, through which superior governments can make evaluations on the effects of the measures adopted by the local governments or institutions in the prevention and control of the virus.

## Conclusion

By combining the theoretical constructions of CGA and CDA and drawing on the 35 press conferences on the COVID-19 outbreak in China, this study explores how the discourse of press conferences is used by the Chinese government as a means of crisis management in an attempt to (re)construct social relations. The findings of the study can be summarized as follows: Firstly, it has been found that four major types of social relations are reproduced discursively in the press conferences on COVID-19 in China, centering around government institutions: relation between government and the general public, relation between government institutions of different levels, relation between government and medical staff, and relation between government and COVID-19 patients. Secondly, on the discursive level, it has been found that the discourse of press conferences on COVID-19 in China has manifested a four-move pattern, with social relations reproduced mainly by a hybridity of four types of discourses: descriptive discourse, instructional discourse, strategic discourse, and evaluative discourse. Thirdly, the hybridity of the discourse of the press conferences on COVID-19 in China can be attributed to several contextual factors, where the dynamics of professional, political, social, and cultural forces are interwoven with each other to contribute to the heterogeneity and complexity of social relations.

By correlating the interdiscursive feature of the discourse of the press conferences on COVID-19 in China with complex social relations and the underpinning socio-cultural dynamics, this study helps to deepen our understanding of how the Chinese government manages crises through a discursive (re)construction of social relations. It can also contribute to our understanding of the complex and dynamic features of discourse, which constitute stratification of language and their underlying professional, social, and cultural contexts of particular world views and tendencies. As this study focuses mainly on the interdiscursive features of press conferences as a whole, insufficient attention has been paid to the heterogeneous features of specific types of discourse that constitute press conferences. Therefore, more detailed studies may be conducted to investigate how different discursive resources are employed to form such discursive phenomena as discourse embedding, discourse mixing, or discourse colonization inside particular types of discourses of press conferences.

## Data availability statement

The original contributions presented in the study are included in the article/supplementary material, further inquiries can be directed to the corresponding author.

## Author contributions

All authors listed have made a substantial, direct, and intellectual contribution to the work, and approved it for publication.

## Funding

This study was supported by National Social Science Fund of China (Grant No. 21BYY093).

## Conflict of interest

The authors declare that the research was conducted in the absence of any commercial or financial relationships that could be construed as a potential conflict of interest.

## Publisher’s note

All claims expressed in this article are solely those of the authors and do not necessarily represent those of their affiliated organizations, or those of the publisher, the editors and the reviewers. Any product that may be evaluated in this article, or claim that may be made by its manufacturer, is not guaranteed or endorsed by the publisher.
